# Fibrinogen-to-Albumin Ratio Predicts Postcontrast Acute Kidney Injury in Patients with Non-ST Elevation Acute Coronary Syndrome after Implantation of Drug-Eluting Stents

**DOI:** 10.1155/2022/9833509

**Published:** 2022-11-23

**Authors:** Yong Qiao, Mingkang Li, Linqing Li, Chengchun Tang

**Affiliations:** Department of Cardiology, Zhongda Hospital, Southeast University, Nanjing, Jiangsu, China

## Abstract

**Background:**

Postcontrast acute kidney injury (PC-AKI) is an adverse reaction to iodinated contrast agents. In this study, we investigated the use of fibrinogen-to-albumin ratio (FAR) as a novel inflammatory marker to track the development and progression of PC-AKI in patients with non-ST elevation acute coronary syndrome (NSTE-ACS) after the implantation of drug-eluting stents (DESs).

**Methods:**

A total of 872 patients with NSTE-ACS were enrolled in this study. PC-AKI was identified when serum creatinine (SCr) levels increased >26.5 mol/L (0.3 mg/dL) or was 1.5 times the baseline level within 48–72 h of exposure to an iodinated contrast agent. The effects of different variables on PC-AKI were evaluated using univariate regression analysis. Multivariate logistic regression analysis was used to determine the independent predictors of PC-AKI. The predictive value of FAR was assessed by estimating the area under the receiver operating characteristic (ROC) curve.

**Results:**

In total, 114 (13.1%) patients developed PC-AKI. The patients with PC-AKI had lower albumin levels (40.5 ± 3.4 vs. 39.0 ± 3.5, *P* < 0.001), higher fibrinogen levels (3.7 ± 0.6 vs. 4.1 ± 0.5, *P* < 0.001), and higher FAR levels (9.2 ± 1.7 vs. 10.5 ± 1.7, *P* < 0.001) than those with non-PC-AKI. There were no significant differences in the preoperative SCr levels between the two groups. After adjusting for confounding factors, FAR was found to be an independent predictor of PC-AKI (OR = 1.478, 95% CI = 1.298–1.684, *P* < 0.001). ROC analysis revealed that for PC-AKI prediction, the area under the curve for FAR was 0.702. The optimum cut-off value of FAR was 10.0, with a sensitivity of 64.9% and a specificity of 69.8%. Moreover, FAR had a higher predictive value for PC-AKI than the Mehran score (0.702 vs. 0.645).

**Conclusion:**

Our study showed that elevated preoperative FAR was closely associated with the development of PC-AKI in patients with NSTE-ACS after implantation of DESs. Therefore, it may be worth monitoring FAR as a guide for using preventive measures to avoid the development of PC-AKI.

## 1. Introduction

Acute coronary syndrome (ACS) is a severe form of coronary artery disease (CAD) and has the highest fatality rate among contemporary noninfectious disorders [1]. Percutaneous coronary intervention (PCI), which emphasizes revascularization strategies, has become an effective treatment for patients with ACS. However, postcontrast acute kidney injury (PC-AKI) is a common complication after PCI in patients with ACS and develops in up to 13.3% of these patients [2]. PC-AKI leads to prolonged hospital stays, increased costs, and increased mortality [3, 4]. Since there are no effective treatments for PC-AKI, it is essential to identify patients at high risk of developing this condition at an early stage and take preventive measures.

To date, several risk models to predict PC-AKI have been developed and used in clinical practice [5, 6]. The most commonly used model for determining the risk of PC-AKI is the Mehran score. However, this score requires information on eight periprocedural risk variables, including presence/history of chronic kidney disease (CKD), hypotension, advanced age, diabetes, anemia, chronic heart failure (CHF), intra-aortic balloon pump (IABP) use, and contrast volume. However, sometimes such information is unavailable before PCI [7].

Evidence has demonstrated that chronic inflammation is a recognized pathological mechanism involved in the development of both CAD and PC-AKI [8, 9]. Fibrinogen, an acute-phase reactive protein involved in coagulation, platelet aggregation, and fibrinolysis, is known to participate in the occurrence and development of inflammatory responses [10, 11]. Albumin is the most abundant protein in the plasma and is a negative acute phase reactant produced by the liver. Decreased albumin levels can increase blood viscosity and endothelial dysfunction, which may contribute to PC-AKI development [12, 13].

Recently, the fibrinogen-to-albumin ratio (FAR) has been identified as a novel inflammation-based risk index and as a prognostic factor for various conditions. Growing evidence has demonstrated that FAR is not only a potential prognostic marker for various cancers [14, 15] but can also predict adverse outcomes in patients with cardiovascular diseases [16, 17]. However, there is insufficient information on the association between FAR and the risk of PC-AKI occurrence in patients with non-ST elevation ACS (NSTE-ACS) who undergo PCI. In light of the aforementioned, this study is aimed at clarifying the relationship between preoperative FAR values and the risk of PC-AKI occurrence in patients with NSTE-ACS implanted with drug-eluting stents (DESs). We believe that our work will help clinicians to evaluate the risk of a patient developing PC-AKI at an early stage and take preventive measures.

## 2. Materials and Methods

### 2.1. Study Population

**Figure 1:**
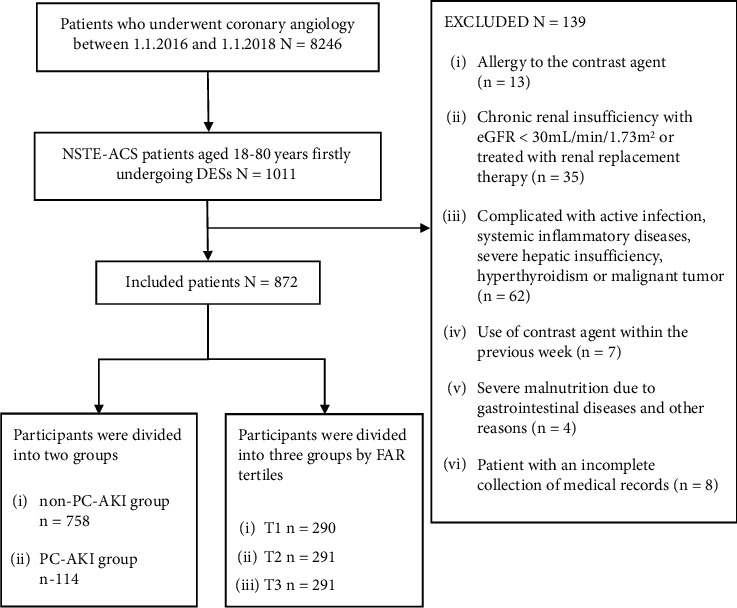
Flow diagram of the patient selection procedure.

This retrospective cohort study was conducted on patients undergoing implantation of DESs at the Zhongda Hospital affiliated with Southeast University between January 2016 and January 2018 (Figure 1). The criteria for admission were (1) patients must be 18–80 years of age, with a definite diagnosis of NSTE-ACS according to the criteria reported by the American College of Cardiology [18]. (2) It must be the patient's first intracoronary procedure for the implantation of DESs. The exclusion criteria were (1) history of allergic reactions to contrast agents; (2) chronic renal insufficiency with estimated glomerular filtration rate (eGFR) < 30 mL/min/1.73 m^2^ or having received renal replacement therapy; (3) complications due to active infections, systemic inflammatory diseases, severe hepatic insufficiency, hyperthyroidism, or presence of malignant tumors; (4) exposure to a contrast agent in the previous week; (5) severe malnutrition due to gastrointestinal diseases or other reasons; (6) having incomplete medical records. The hospital ethics committee approved this study and written informed consent was obtained from all patients.

### 2.2. Data Collection, Definitions, and Coronary Interventions

The baseline characteristics of the patients were obtained from the medical record systems by clinicians blinded to the study protocol. Fasting blood samples of the patients were obtained within 24 h of admission and stored at -80°C until tested. A Stago automatic analyzer with the STA fibrinogen kit (Diagnostica Stago, Taverny, France) was used to measure the concentrations of fibrinogen in preoperative plasma samples. An automatic chemical analyzer (AU5400, Olympus, Japan) was used to measure the preoperative levels of serum albumin using the bromocresol green dye method. Serum creatinine (SCr) levels were measured before and after PCI over a span of 2–3 days. The Department of Laboratory Medicine at Zhongda Hospital performed all laboratory-based measurements. Preoperative FAR was calculated as a percentage using the following equation: [serum fibrinogen (g/L)/albumin (g/L)]^∗^ 100. The Chronic Kidney Disease Epidemiology Collaboration (CKD-EPI) equation was used to determine the estimated glomerular filtration rate (eGFR) [19]. Transthoracic echocardiography (Philips Medical Systems, Amsterdam, Netherlands) was used regularly with the Simpson apical biplane technique to estimate the preoperative left ventricular ejection fraction (LVEF). The Mehran score risk model with eight variables, namely, hypotension (5 points), CHF (5 points), IABP use (5 points), age > 75 years (4 points), eGFR < 60 mL/min/1.73m^2^ (range: 40 > 60, 2 points; 20 > 40, 4 points; <20, 6 points), diabetes (3 points), anemia (3 points), and contrast volume (1 point for each 100 mL) was calculated for each patient as the sum of the points obtained by the presence of the risk factors mentioned for that patient [20].

According to the 2018 recommendations of the European Society of Urogenital Radiology, PC-AKI is defined as an increase in SCr levels > 26.5 mol/L (0.3 mg/dL), or to a value 1.5 times the baseline value, in the 48–72 h following PCI [21]. A patient was diagnosed with hypertension if the patient was receiving antihypertensive treatment at that time or if the patient's systolic blood pressure (SBP) was ≥140 mmHg or/and diastolic blood pressure (DBP) was ≥90 mmHg for three or more consecutive readings on different days. Patients were diagnosed as being diabetic according to the latest guidelines of the American Diabetes Association [22]. Anemia was diagnosed if an adult nonpregnant female patient had a hemoglobin concentration < 12.0 g/dL or an adult male patient had a hemoglobin concentration < 13.0 g/dL. CHF was diagnosed if the patient exhibited symptoms/signs of congestive heart disease as per the New York Heart Association (functional class > II). Patients were diagnosed with the three-vessel disease if they exhibited angiographic stenosis of ≥50% in all three major coronary arteries, including the left anterior descending, circumflex, and right coronary arteries, with or without the involvement of the left main artery.

All PCI procedures were performed in the cardiac catheterization laboratory by experienced specialists. The Seldinger puncture method was used in the operation with the radial or femoral artery as the site of the puncture. All stents used in the PCI procedures were DESs. Before surgery, all patients received loading doses of dual antiplatelet aggregation treatment (300 mg of aspirin, 300 mg of clopidogrel, or 180 mg of ticagrelor). The interventional physician determined the postoperative medication regimen according to the clinical condition of each patient.

### 2.3. Groups

Patients were grouped according to whether or not they developed PC-AKI. There were 114 patients in the PC-AKI group and 758 patients in the non-PC-AKI group. Additionally, we divided the patients into three groups based on their FAR according to tertiles (group T1 with FAR ≤ 8.57, *n* = 290; group T2 with FAR = 8.58–10.09, *n* = 291; and group T3 with FAR ≥ 10.10, *n* = 291).

### 2.4. Statistical Analysis

We used the IBM SPSS statistics software for macOS, version 26 (IBM Corp., Armonk, N.Y., USA) and GraphPad Prism 9 for macOS, version 9.1.1 (GraphPad Software, LLC., San Diego, USA) for data analyses. Normally distributed data were expressed as mean ± standard deviation and intergroup comparisons for such data were performed using independent sample *t*-tests or one-way ANOVAs. Nonnormally distributed data were represented as median values and interquartile ranges; the rank-sum test was used for the analysis of these data. Count data were expressed in percentages (%) and analyzed using either the chi-square test or Fisher's exact test. We also used Spearman's and Pearson's correlation analyses to evaluate the relationships between FAR and other clinical factors. Univariate and multivariate logistic regression analyses combined with the backward stepwise method (probability for stepwise: entry 0.05, removal 0.10) was used to determine the risk factors for PC-AKI. The receiver operating characteristic (ROC) curve was used to evaluate the predictive value of preoperative FAR values for the occurrence of PC-AKI. All tests were two-tailed, and a *P* < 0.05 was considered statistically significant.

## 3. Results

**Figure 2:**
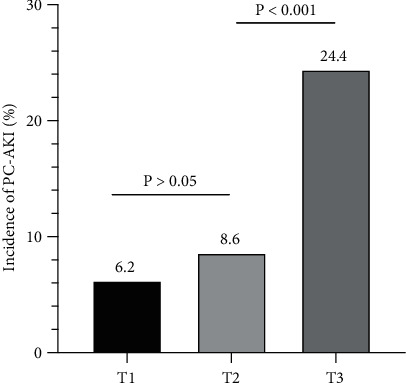
The incidence of postcontrast acute kidney injury (PC-AKI) in different FAR (fibrinogen–albumin ratio) groups.

This study involved 872 patients with NSTE-ACS, of which 63.5% were men; the average age of this patient pool was 64.6 ± 9.3 years and the average FAR was 9.4 ± 1.8. Of these patients, 114 (13.1%) developed PC-AKI after PCI.  Table 1 shows that the PC-AKI group had significantly higher FAR values than the non-PC-AKI group (10.5 ± 1.7 vs. 9.2 ± 1.7, *P* < 0.001). The patients who developed PC-AKI were less hydrated, had lower levels of hemoglobin and albumin, and also had lower eGFR values than those in the non-PC-AKI group. In addition, there were more women in the PC-AKI group than in the non-PC-AKI group. Moreover, more patients in the PC-AKI group suffered from anemia and CHF, and used diuretics than in the non-PC-AKI group. The patients in the PC-AKI group also had higher levels of fasting blood glucose, D-dimer, and fibrinogen, as well as higher hemoglobin A1c (HbA1c) and Mehran scores than those in the non-PC-AKI group. There were no significant differences (*P* > 0.05) between the groups in terms of age; body mass index (BMI); incidence of stroke, hypertension, diabetes, smoking, CKD, and non-ST elevation myocardial infarction (NSTEMI); values of SBP and DBP; leukocyte counts; platelet counts; levels of SCr, uric acid, triglyceride, total cholesterol (TC), high-density lipoprotein cholesterol (HDL-C), and low-density lipoprotein and cholesterol (LDL-C); prothrombin time (PT) and activated partial thromboplastin time (APTT); LVEF; incidence of left main disease or three-vessel disease; numbers of stents; contrast volumes; and usage of aspirin, clopidogrel/ticagrelor, angiotensin-converting enzyme inhibitor (ACEI)/angiotensin receptor blocker (ARB), *β*-blockers, and statins. However, the incidence of PC-AKI increased in a stepwise manner with each succeeding tertile of FAR values (6.2%, 8.6%, and 24.4%, respectively, *P* < 0.001) (Figure 2). As shown in Table 2, there were statistically significant differences among the three FAR groups in terms of sex; age; incidence of anemia, NSTEMI, and CHF; leukocyte counts; levels of hemoglobin and SCr; eGFR; levels of HDL-C, D-dimer, fibrinogen, albumin, and HbA1c; incidence of three-vessel disease; hydration levels; and use of *β*-blockers and diuretics. No statistically significant differences were observed between the three groups in the other variables.

As shown in Table 3, the Spearman and Pearson correlation analyses revealed that FAR was positively correlated with age, SCr levels, and HbA1c levels, while negatively correlated with hemoglobin levels, eGFR values, and HDL-C levels. Univariate logistic regression analysis showed that FAR (OR = 1.535, 95% CI = 1.361–1.731, *P* < 0.001); sex; anemia; CHF; levels of hemoglobin, platelets, SCr, D-dimer, fibrinogen, and albumin; eGFR; LVEF; hydration levels; and use of diuretics were risk factors for PC-AKI (Supplementary Table 1). As summarized in Table 4, multivariate logistic regression analysis revealed that FAR (OR = 1.478, 95% CI = 1.298–1.684, *P* < 0.001), anemia, CHF, TC, and use of diuretics were independent risk factors for PC-AKI after adjusting for confounding factors (including sex; age; BMI; presence of hypertension, diabetes, anemia, CKD, NSTEMI, and CHF; SBP; leukocyte counts; levels of hemoglobin, platelets, fasting blood glucose, SCr, TC, HDL-C, LDL-C, D-dimer, fibrinogen, albumin, and HbA1c; eGFR; LVEF; the presence of three-vessel disease; contrast volume; hydration levels; and use of ACEI/ARB, *β*-blockers, statins, and diuretics). We also found that age and HDL-C were independent protective factors.

**Figure 3:**
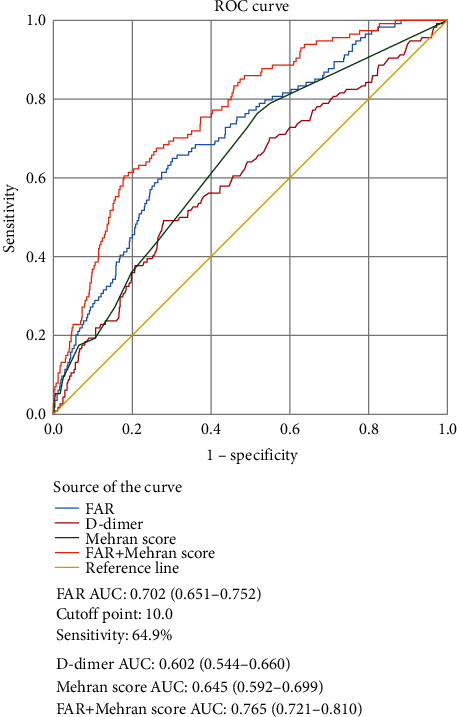
Receiver operating characteristic (ROC) curve analysis for the fibrinogen-to-albumin ratio (FAR) and Mehran score in predicting postcontrast acute kidney injury (PC-AKI).

**Figure 4:**
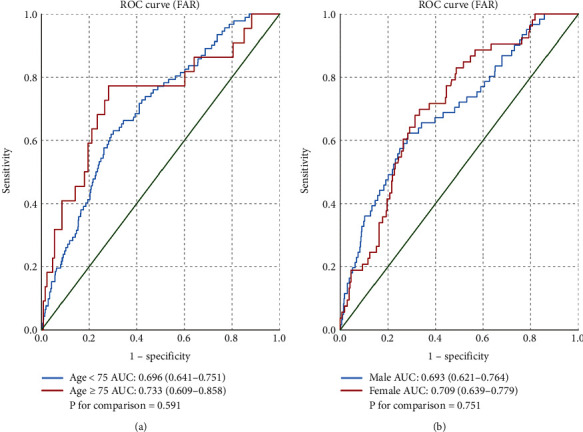
The impact of the fibrinogen-to-albumin ratio (FAR) in predicting the incidence of postcontrast acute kidney injury (PC-AKI) in the (a) age and (b) sex subgroups.

The ROC curve analysis indicates that FAR provides the highest predictive value of the three single parameters (0.702, 0.602, and 0.645, respectively) (Figure 3). The optimum cut-off point of FAR was 10.0, with a sensitivity of 64.9% and specificity of 69.8%. In addition, when FAR was included in the Mehran risk model for predicting PC-AKI, the area under the curve (AUC) increased from 0.645 to 0.765. As shown in Figure 4, there was no statistically significant difference in the ability of FAR to predict PC-AKI between the <75 years and ≥ 75 years age groups (0.696 vs. 0.733, *P* > 0.05). Meanwhile, the AUC for FAR in predicting PC-AKI in men was similar to that in women (0.693 vs. 0.709, *P* > 0.05).

## 4. Discussion

This study investigated if preoperative FAR values could be used to predict the development of PC-AKI in patients with NSTE-ACS who had undergone surgery for the implantation of DESs. We found that preoperative FAR was an independent risk factor for PC-AKI in these patients. Notably, FAR had a higher predictive value than the Mehran score in these patients; it is possible that higher preoperative FAR levels may be a potentially valuable predictor of PC-AKI.

In our study, the Mehran score effectively identified patients with PC-AKI. This was expected as it represents a well-tested and reliable score for predicting PC-AKI. However, combining FAR values with the other variables in the Mehran score improved its predictive power. This addition of a simple and easy-to-use parameter, such as FAR, to the Mehran score can be extremely valuable in clinical medicine. Evidence has shown that FAR, a value derived from the levels of albumin and fibrinogen in plasma, is strongly associated with cardiovascular events. Li et al. have reported that FAR is independently associated with CAD severity and can be used as a prognostic indicator for such conditions. In addition, FAR values could also be used to improve risk stratification in patients with NSTE-ACS [17]. A prospective cohort study involving 562 patients showed that FAR was an independent risk factor for all-cause mortality in patients with ST-segment elevation myocardial infarction (STEMI) and multivessel disease and that it helped in guiding clinical strategies [23]. A nonlinear relationship between FAR and in-hospital mortality among critically ill patients with AKI was also observed by Xia et al. [24]. However, to our knowledge, no study has yet focused on the relationship between preoperative FAR and PC-AKI in patients with NSTE-ACS. Our results fill this gap by demonstrating that high preoperative FAR values are independently associated with an increased risk of PC-AKI in patients with NSTE-ACS implanted with DESs. Additionally, we determined that the best cut-off value of FAR for predicting PC-AKI was 10.0, with a specificity of 69.8% and a sensitivity of 64.9%. ROC curve analysis revealed that the AUC for FAR in predicting PC-AKI was 0.602. With the addition of FAR, the predictive values of the Mehran models for PC-AKI improved, suggesting that patients with elevated FAR may represent a previously unrecognized high-risk group.

Previous work has shown that anemia is associated with an increased risk of PC-AKI (pooled OR = 1.82, 95% CI = 1.27–2.61) [25]. The proportion of patients with anemia in the PC-AKI group was significantly higher than in the non-PC-AKI group. Consistent with the previous study, our multivariate logistic regression analysis showed that anemia was an independent risk factor for developing PC-AKI (OR = 1.750, 95% CI = 1.031–2.968). These results underscore the importance of treating anemia as a crucial step for preventing PC-AKI. Interestingly, the patients in the PC-AKI group had significantly higher concentrations of D-dimer than those in the non-PC-AKI group. Previous studies have shown that elevated D-dimer levels are valuable biomarkers of renal diseases. Our results are similar to those of Lin et al., who found that elevated D-dimer levels are a strong predictor of PC-AKI after PCI, with an AUC of 0.729 [26].

Additionally, we noted that age was negatively associated with the development of PC-AKI, which differed from our prior expectations that aging would increase susceptibility to AKI. In a retrospective study by Xu et al., the relationship between age and AKI in 47,012 adult patients [27] was found to be a “U-shaped” curve. In other words, the incidence of AKI was negatively correlated with age in adults <75 years but was positively correlated with age in adults >75 years. Most patients included in this study were < 75 years, which partly explains our results. We performed subgroup analyses to determine whether FAR had a high prognostic value in specific subgroups; however, our results showed that there were no statistically significant differences in the ability of FAR to predict PC-AKI incidence within the age and sex subgroups. This result inversely demonstrated that FAR is a robust predictor of PC-AKI incidence.

Although the pathogenesis of PC-AKI has not yet been fully elucidated, its occurrence may be related to inflammatory activation, renal medullary hypoxia, increased blood viscosity, and/or oxygen-free radical damage [28, 29]. The mechanisms underlying the association between high FAR values and the incidence of PC-AKI likely depend on the opposite roles played by albumin and fibrinogen in inflammation. Since albumin is a negative acute-phase reactant, it reduces acute and chronic inflammatory states [30]. Additionally, albumin has antioxidant properties that aid in scavenging oxygen free radicals in plasma [31], and reductions in albumin levels increase blood viscosity, resulting in endothelial dysfunction [32, 33]. In contrast, fibrinogen is a positive acute-phase reactant involved in systemic inflammatory responses [34]. Elevated fibrinogen levels increase blood viscosity resulting in increased endothelial shear stress and impaired endothelial function [35, 36].

Although our study reports some important and novel results, it has several limitations. (1) As a retrospective observational study, the risks of bias and residual confounding effects could not be entirely excluded although we attempted to adjust for such issues. (2) No data on the perioperative hydration volumes were available although these values may have affected the incidence of PC-AKI. (3) Due to the retrospective design of our study, it is impossible for us to demonstrate a causal relationship between FAR and PC-AKI. (4) Changes in FAR levels during hospitalization were not monitored and it is still unclear if reductions in FAR values can significantly improve clinical outcomes. (5) This study lacks data on traditional hematological markers, such as neutrophil gelatinase-associated lipocalin, cystatin C, or myeloperoxidase as references.

## 5. Conclusion

Preoperative FAR values seem to be reliable markers for identifying the risk of PC-AKI development in patients with NSTE-ACS who are undergoing implantation of DESs. Further research using larger sample sizes and prospective randomized controlled trials are needed before these conclusions can be applied to regular clinical practice.

## Figures and Tables

**Table 1:** Baseline characteristics of patients with and without PC-AKI.

Variable	Total patients	Non-PC-AKI	PC-AKI	*P* value
*N*	872	758	114	
Male (*n*, %)	554 (63.5)	493 (65.0)	61 (53.5)	0.017
Age (years)	64.6 ± 9.3	64.6 ± 9.1	64.5 ± 10.3	0.904
BMI (kg/m^2^)	25.1 ± 3.1	25.0 ± 3.1	25.5 ± 3.3	0.143
Previous stroke (*n*, %)	186 (21.3)	159 (21.0)	27 (23.7)	0.510
Hypertension (*n*, %)	627 (71.9)	539 (71.1)	88 (77.2)	0.178
Diabetes (*n*, %)	265 (30.4)	222 (29.3)	43 (37.7)	0.068
Smoking (*n*, %)	244 (28.0)	216 (28.5)	28 (24.6)	0.383
Anemia (*n*, %)	128 (14.7)	98 (11.9)	30 (26.3)	<0.001
CKD (*n*, %)	20 (2.3)	16 (2.1)	4 (3.5)	0.318
NSTEMI (*n*, %)	155 (17.8)	128 (16.9)	27 (23.7)	0.077
CHF (*n*, %)	84 (9.6)	59 (7.8)	25 (21.9)	<0.001
SBP (mmHg)	136.7 ± 19.3	136.3 ± 19.1	139.1 ± 20.2	0.141
DBP (mmHg)	77.1 ± 12.3	77.1 ± 12.3	77.3 ± 12.6	0.883
Leukocyte (10^9^/L)	6.5 (5.5, 7.8)	6.4 (5.5, 7.7)	6.8 (5.6, 8.2)	0.090
Hemoglobin (g/L)	138.9 ± 15.2	139.6 ± 15.0	134.3 ± 15.6	0.001
Platelet (10^9^/L)	198 (165, 237)	196 (165, 235)	212 (168, 249)	0.060
Fasting blood glucose (mmol/L)	5.8 (5.2, 7.3)	5.9 (5.3, 7.4)	6.0 (5.2, 8.2)	0.023
SCr (*μ*mol/L)	75 (65, 88)	75 (64, 87)	79 (67, 95)	0.056
eGFR (mL/min/1.73 m^2^)	87.0 (72.2, 99.1)	87.7 (73.6, 99.3)	79.9 (62.7, 97.6)	0.007
Uric acid (*μ*mol/L)	350 (293, 412)	350 (294, 409)	349 (284, 420)	0.851
Triglyceride (mmol/L)	1.4 (1.0, 2.1)	1.4 (1.0, 2.1)	1.6 (1.0, 2.2)	0.162
TC (mmol/L)	4.4 (3.7, 5.2)	4.4 (3.7, 5.2)	4.5 (3.8, 5.5)	0.121
HDL-C (mmol/L)	1.1 (1.0, 1.3)	1.1 (1.0, 1.3)	1.1 (0.9, 1.3)	0.227
LDL-C (mmol/L)	2.7 (2.1, 3.3)	2.6 (2.1, 3.3)	2.8 (2.1, 3.5)	0.197
PT (*s*)	11.6 ± 2.9	11.4 ± 1.7	11.5 ± 1.9	0.551
APTT (*s*)	31.0 ± 4.8	30.9 ± 5.2	31.1 ± 3.1	0.714
D-dimer (*μ*g/L)	92 (52, 151)	89 (50, 143)	122 (64, 181)	<0.001
Fibrinogen (g/L)	3.8 ± 0.6	3.7 ± 0.6	4.1 ± 0.5	<0.001
Albumin (g/L)	40.3 ± 3.5	40.5 ± 3.4	39.0 ± 3.5	<0.001
FAR (%)	9.4 ± 1.8	9.2 ± 1.7	10.5 ± 1.7	<0.001
Mehran score	4 (1, 5)	3 (1, 5)	5 (3, 7)	<0.001
HbA1c (%)	6.0 (5.6, 6.8)	6.0 (5.6, 6.8)	6.2 (5.7, 7.2)	0.027
LVEF (%)	67 (62, 72)	67 (62, 72)	68 (60, 72)	0.585
Procedural characteristics				
Left main disease (*n*, %)	61 (7.0)	55 (7.3)	6 (5.3)	0.437
Three-vessel disease (*n*, %)	337 (38.7)	287 (37.9)	50 (43.9)	0.220
Number of stents	1.5 ± 0.7	1.5 ± 0.7	1.5 ± 0.6	0.916
Contrast volume (mL)	100 (100, 100)	100 (100, 100)	100 (100, 200)	0.181
Hydration (*n*, %)	714 (81.9)	639 (84.3)	75 (65.8)	<0.001
Medication use (*n*, %)				
Aspirin	857 (98.3)	744 (98.2)	113 (99.1)	0.707
Clopidogrel/ticagrelor	870 (99.8)	757 (99.9)	113 (99.1)	0.245
ACEI/ARB	485 (55.6)	421 (55.5)	64 (56.1)	0.904
*β*-Blockers	683 (78.3)	592 (78.1)	91 (79.8)	0.677
Statin	869 (99.7)	756 (99.7)	113 (99.1)	0.344
Diuretics	96 (11.0)	69 (9.1)	27 (23.7)	<0.001

PC-AKI: postcontrast acute kidney injury; BMI: body mass index; CKD: chronic kidney disease; NSTEMI: non-ST elevation myocardial infarction; CHF: congestive heart failure; SBP: systolic blood pressure; DBP: diastolic blood pressure; SCr: serum creatinine; eGFR: estimated glomerular filtration rate; TC: total cholesterol; HDL-C: high-density lipoprotein cholesterol; LDL-C: low-density lipoprotein cholesterol; PT: prothrombin time; APTT: activated partial thromboplastin time; FAR: fibrinogen-to-albumin ratio; HbA1c: hemoglobin A1c; LVEF: left ventricular ejection fraction; ACEI: angiotensin-converting enzyme inhibitor; ARB: angiotensin receptor blocker.

**Table 2:** Baseline characteristics among three groups.

Variable	T 1	T 2	T 3	*P* value
*N*	290	291	291	
Male (*n*, %)	207 (71.4)	170 (58.4)	177 (60.8)	0.003
Age (years)	63.0 ± 9.1	65.0 ± 9.3	65.8 ± 9.3	0.001
BMI (kg/m^2^)	25.1 ± 3.1	24.9 ± 3.2	25.2 ± 3.1	0.698
Prior stroke (*n*, %)	64 (22.1)	56 (19.2)	66 (22.7)	0.558
Hypertension (*n*, %)	203 (70.0)	209 (71.8)	215 (73.9)	0.581
Diabetes (*n*, %)	75 (25.9)	96 (33.0)	94 (32.3)	0.120
Smoking (*n*, %)	86 (29.7)	84 (28.9)	74 (25.4)	0.483
Anemia (*n*, %)	27 (9.3)	39 (13.4)	62 (21.3)	<0.001
CKD (*n*, %)	9 (3.1)	4 (1.4)	7 (2.4)	0.375
NSTEMI (*n*, %)	47 (16.2)	35 (12.0)	73 (25.1)	<0.001
CHF (*n*, %)	15 (5.2)	26 (8.9)	43 (14.8)	<0.001
SBP (mmHg)	137.1 ± 19.9	137.0 ± 19.3	135.9 ± 18.6	0.713
DBP (mmHg)	78.0 ± 12.4	76.7 ± 12.0	76.8 ± 12.6	0.366
Leukocyte (10^9^/L)	6.2 (5.2, 7.3)	6.5 (5.6, 7.9)	6.8 (5.8, 8.1)	<0.001
Hemoglobin (g/L)	143.1 ± 13.6	138.0 ± 15.4	135.6 ± 15.5	<0.001
Platelet (10^9^/L)	198 (164, 232)	196 (163, 237)	199 (170, 243)	0.224
Fasting blood glucose (mmol/L)	5.8 (5.2, 6.9)	5.8 (5.2, 7.2)	5.9 (5.2, 7.9)	0.564
SCr (*μ*mol/L)	74 (63, 86)	74 (64, 86)	78 (67, 91)	0.006
eGFR (mL/min/1.73 m^2^)	91.0 (77.8, 102.3)	86.2 (72.3, 98.3)	81.3 (68.5, 95.9)	<0.001
Uric acid (*μ*mol/L)	350 (295, 406)	335 (288, 412)	364 (297, 417)	0.101
Triglyceride (mmol/L)	1.5 (1.0, 2.2)	1.4 (1.0, 2.0)	1.5 (1.1, 2.1)	0.471
TC (mmol/L)	4.5 (3.7, 5.1)	4.4 (3.7, 5.2)	4.5 (3.8, 5.2)	0.585
HDL-C (mmol/L)	1.1 (1.0, 1.3)	1.1 (0.9, 1.3)	1.1 (0.9, 1.2)	0.020
LDL-C (mmol/L)	2.7 (2.1, 3.3)	2.6 (2.0, 3.3)	2.7 (2.1, 3.4)	0.422
PT (*s*)	11.4 ± 1.1	11.5 ± 2.5	11.5 ± 1.3	0.825
APTT (*s*)	30.7 ± 4.1	31.1 ± 6.7	31.0 ± 3.7	0.622
D-dimer (*μ*g/L)	71 (43, 119)	95 (55, 153)	113 (66, 181)	<0.001
Fibrinogen (g/L)	3.1 ± 0.4	3.8 ± 0.3	4.4 ± 0.4	<0.001
Albumin (g/L)	41.9 ± 3.2	40.6 ± 3.2	38.4 ± 3.1	<0.001
FAR (%)	7.5 ± 0.9	9.3 ± 0.4	11.4 ± 0.9	<0.001
Mehran score	1 (1, 4)	4 (1, 5)	4 (1, 6)	<0.001
HbA1c (%)	6.0 (5.5,6.5)	6.1 (5.7, 6.9)	6.1 (5.6, 7.1)	0.008
LVEF (%)	68 (63, 72)	67 (63, 72)	67 (60, 73)	0.617
Procedural characteristics				
Left main disease (*n*, %)	16 (5.5)	19 (6.5)	26 (8.9)	0.252
Three-vessel disease (*n*, %)	99 (34.1)	108 (37.1)	130 (44.7)	0.027
Number of stents	1.5 ± 0.6	1.5 ± 0.7	1.5 ± 0.6	0.992
Contrast volume (mL)	100 (100, 100)	100 (100, 100)	100 (100, 100)	0.938
Hydration (*n*, %)	255 (87.9)	237 (81.4)	222 (76.3)	0.001
Medication use (*n*, %)				
Aspirin	285 (98.3)	284 (97.6)	288 (99.0)	0.433
Clopidogrel/ticagrelor	289 (99.7)	291 (100)	290 (99.7)	0.443
ACEI/ARB	155 (53.4)	157 (54.0)	173 (59.5)	0.271
*β*-Blockers	215 (74.1)	227 (78.0)	241 (82.8)	0.039
Statin	288 (99.3)	290 (99.7)	291 (100)	0.248
Diuretics	20 (6.9)	31 (10.7)	45 (15.5)	0.004
PC-AKI (*n*, %)	18 (6.2)	25 (8.6)	71 (24.4)	<0.001

PC-AKI: postcontrast acute kidney injury; BMI: body mass index; CKD: chronic kidney disease; NSTEMI: non-ST elevation myocardial infarction; CHF: congestive heart failure; SBP: systolic blood pressure; DBP: diastolic blood pressure; SCr: serum creatinine; eGFR: estimated glomerular filtration rate; TC: total cholesterol; HDL-C: high-density lipoprotein cholesterol; LDL-C: low-density lipoprotein cholesterol; PT: prothrombin time; APTT: activated partial thromboplastin time; FAR: fibrinogen-to-albumin ratio; HbA1c: hemoglobin A1c; LVEF: left ventricular ejection fraction; ACEI: angiotensin-converting enzyme inhibitor; ARB: angiotensin receptor blocker.

**Table 3:** Correlation between FAR and clinical factors.

Variables	Correlation coefficient (*r*)	*P* value
Age	0.158	< 0.001
BMI	-0.019	0.057
SBP	-0.014	0.687
DBP	-0.035	0.297
Hemoglobin	-0.203	< 0.001
Fasting blood glucose	0.023	0.492
SCr	0.099	0.003
eGFR	-0.174	< 0.001
Uric acid	0.057	0.093
HbA1c	0.078	0.022
Triglyceride	-0.014	0.681
TC	-0.013	0.705
HDL-C	-0.113	0.001
LDL-C	0.029	0.392

FAR: fibrinogen-to-albumin ratio; BMI: body mass index; SBP: systolic blood pressure; DBP: diastolic blood pressure; SCr: serum creatinine; eGFR: estimated glomerular filtration rate; HbA1c: hemoglobin A1c; TC: total cholesterol; HDL-C: high-density lipoprotein cholesterol; LDL-C: low-density lipoprotein cholesterol.

**Table 4:** Multivariate logistic analysis for predicting PC-AKI after DESs implantation.

Variables	OR	95% CI	*P* value
FAR	1.478	1.298–1.684	< 0.001
Female	1.547	0.988–2.423	0.056
Age	0.960	0.934–0.986	0.003
Anemia	1.750	1.031–2.968	0.038
CHF	1.927	1.033–3.595	0.039
eGFR	0.986	0.974–0.999	0.179
TC	1.965	1.207–3.198	0.007
HDL-C	0.355	0.163–0.774	0.009
LDL-C	0.606	0.344–1.067	0.083
Diuretics	2.152	1.197–3.869	0.010

FAR: fibrinogen-to-albumin ratio; CHF: congestive heart failure; eGFR: estimated glomerular filtration rate; TC: total cholesterol; HDL-C: high-density lipoprotein cholesterol; LDL-C: low-density lipoprotein cholesterol.

## Data Availability

On reasonable request, data of this article may be obtained from the corresponding author.

## Supplementary Materials

Supplementary Materials Supplementary Table 1: univariate logistic analysis for predicting PC-AKI after DESs implantation.
