# Supplementary material for: Fibrinogen-to-Albumin Ratio Predicts Postcontrast Acute Kidney Injury in Patients with Non-ST Elevation Acute Coronary Syndrome after Implantation of Drug-Eluting Stents
Source: J Renin Angiotensin Aldosterone Syst. 2022 Nov 23;2022:9833509. doi: 10.1155/2022/9833509 (PMC9711978; doi:10.1155/2022/9833509)
Supplement: Supplementary Materials — Supplementary Table 1: univariate logistic analysis for predicting PC-AKI after DESs implantation. [file 9833509.f1.docx]

**Supplementary Table 1:** Univariate logistic analysis for predicting PC-AKI after DESs implantation

| Variables | OR | 95%CI | P-value |
| --- | --- | --- | --- |
| FAR | 1.535 | 1.361–1.731 | < 0.001 |
| Female | 1.616 | 1.087–2.405 | 0.018 |
| Age | 0.999 | 0.978–1.020 | 0.895 |
| BMI | 1.047 | 0.984–1.114 | 0.144 |
| Previous stroke | 1.169 | 0.734–1.863 | 0.511 |
| Hypertension | 1.375 | 0.864–2.189 | 0.179 |
| Diabetes | 1.462 | 0.971–2.203 | 0.069 |
| Smoking | 0.817 | 0.518–1.287 | 0.384 |
| Anemia | 2.405 | 1.507–3.840 | < 0.001 |
| CKD | 1.686 | 0.554–5.137 | 0.160 |
| NSTEMI | 1.527 | 0.953–2.448 | 0.078 |
| CHF | 3.328 | 1.984–5.582 | < 0.001 |
| SBP | 1.008 | 0.998–1.018 | 0.141 |
| DBP | 1.001 | 0.985–1.017 | 0.883 |
| Leukocyte | 1.087 | 0.974–1.212 | 0.136 |
| Hemoglobin | 0.977 | 0.964–0.990 | 0.001 |
| Platelet | 1.004 | 1.000–1.008 | 0.043 |
| Fasting blood glucose | 1.039 | 0.971–1.112 | 0.265 |
| SCr | 1.010 | 1.000–1.019 | 0.041 |
| eGFR | 0.985 | 0.976–0.995 | 0.005 |
| Uric acid | 1.000 | 0.998–1.002 | 0.978 |
| Triglyceride | 1.066 | 0.945–1.202 | 0.300 |
| TC | 1.155 | 0.987–1.352 | 0.073 |
| HDL-C | 0.556 | 0.294–1.049 | 0.070 |
| LDL-C | 1.192 | 0.981–1.448 | 0.077 |
| PT | 1.028 | 0.938–1.126 | 0.560 |
| APTT | 1.007 | 0.971–1.044 | 0.714 |
| D-dimer | 1.003 | 1.001–1.004 | 0.001 |
| Fibrinogen | 2.800 | 1.964–3.993 | < 0.001 |
| Albumin | 0.881 | 0.830–0.935 | < 0.001 |
| HbA1c | 1.118 | 0.978–1.278 | 0.103 |
| LVEF | 0.979 | 0.964–0.995 | 0.009 |
| Left main disease | 0.710 | 0.298–1.689 | 0.439 |
| Three-vessel disease | 1.282 | 0.861–1.909 | 0.221 |
| Number of stents | 0.984 | 0.729–1.328 | 0.916 |
| Contrast volume | 1.004 | 0.998–1.011 | 0.184 |
| Hydration | 0.358 | 0.232–0.553 | < 0.001 |
| Aspirin | 2.126 | 0.277–16.326 | 0.468 |
| Clopidogrel/ Ticagrelor | 0.149 | 0.009–2.403 | 0.180 |
| ACEI/ARB | 1.025 | 0.689–1.524 | 0.904 |
| β-blockers | 1.109 | 0.681–1.809 | 0.677 |
| Statin | 0.299 | 0.027–3.324 | 0.326 |
| Diuretics | 3.099 | 1.884–5.097 | < 0.001 |

PC-AKI: post-contrast acute kidney injury, BMI: body mass index, CKD: chronic kidney disease, NSTEMI: non-ST elevation myocardial infarction, CHF: congestive heart failure, SBP: systolic blood pressure, DBP: diastolic blood pressure, SCr: serum creatinine, eGFR: estimated glomerular filtration rate, TC: total cholesterol, HDL-C: high-density lipoprotein cholesterol, LDL-C: low-density lipoprotein cholesterol, PT: prothrombin time, APTT: activated partial thromboplastin time, FAR: fibrinogen-to-albumin ratio, HbA1c: hemoglobin A1c, LVEF: left ventricular ejection fraction, ACEI: angiotensin-converting enzyme inhibitor, ARB: angiotensin receptor blocker.
